# Penta­decyl­ammonium methyl sulfate

**DOI:** 10.1107/S1600536811011226

**Published:** 2011-04-07

**Authors:** Lijun Zhang, Youying Di, Wenyan Dan

**Affiliations:** a College of Chemistry and Chemical Engineering, Liaocheng University, Shandong 252059, People’s Republic of China

## Abstract

In the crystal of the title compound, C_15_H_34_N^+^·CH_3_SO_4_^−^, the cations and anions are joined together *via* strong N—H⋯O hydrogen bonds into layers parallel to (001).

## Related literature

Long-chain *n*-alkyl­ammonium halides are widely used as surfacta­nts (Aratono *et al.*, 1998 ▶; Tornblom *et al.*, 2000 ▶) and as models for biological membranes (Ringsdorf *et al.*, 1988 ▶). For solid-solid phase transitions in *n*-alkyl­ammonium chlorides, see: Terreros *et al.* (2000 ▶).
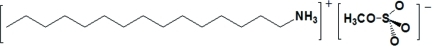


## Experimental

### 

#### Crystal data

C_15_H_34_N^+^·CH_3_O_4_S^−^
*M* *_r_* = 339.53
Monoclinic, 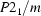

*a* = 5.4260 (5) Å
*b* = 7.4981 (6) Å
*c* = 24.376 (2) Å
β = 93.557 (1)°
*V* = 989.83 (15) Å^3^
*Z* = 2
Mo *K*α radiation
μ = 0.18 mm^−1^
*T* = 298 K
0.31 × 0.30 × 0.28 mm

#### Data collection

Siemens SMART CCD area-detector diffractometer
Absorption correction: multi-scan (*SADABS*; Sheldrick, 1996 ▶) *T*_min_ = 0.946, *T*_max_ = 0.951
5243 measured reflections
1880 independent reflections
1025 reflections with *I* > 2σ(*I*)
*R*_int_ = 0.062

#### Refinement

*R*[*F* ^2^ > 2σ(*F* ^2^)] = 0.074
*wR*(*F* ^2^) = 0.228
*S* = 1.06
1880 reflections
130 parameters
H-atom parameters constrained
Δρ_max_ = 0.43 e Å^−3^
Δρ_min_ = −0.24 e Å^−3^

### 

Data collection: *SMART* (Siemens, 1996 ▶); cell refinement: *SAINT* (Siemens, 1996 ▶); data reduction: *SAINT*; program(s) used to solve structure: *SHELXS97* (Sheldrick, 2008 ▶); program(s) used to refine structure: *SHELXL97* (Sheldrick, 2008 ▶); molecular graphics: *SHELXTL* (Sheldrick, 2008 ▶); software used to prepare material for publication: *SHELXTL*.

## Supplementary Material

Crystal structure: contains datablocks I, global. DOI: 10.1107/S1600536811011226/ru2002sup1.cif

Structure factors: contains datablocks I. DOI: 10.1107/S1600536811011226/ru2002Isup2.hkl

Additional supplementary materials:  crystallographic information; 3D view; checkCIF report

## Figures and Tables

**Table 1:** Hydrogen-bond geometry (Å, °)

*D*—H⋯*A*	*D*—H	H⋯*A*	*D*⋯*A*	*D*—H⋯*A*
N1—H1*A*⋯O2^i^	0.89	2.03	2.857 (6)	155
N1—H1*B*⋯O3^ii^	0.89	2.03	2.911 (3)	169

Symmetry codes: (i) 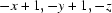; (ii) 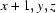.

**Fig. 1.**
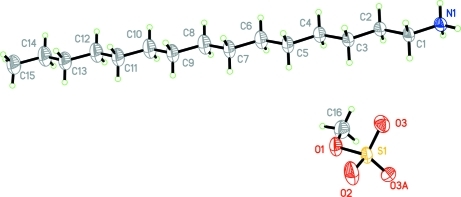
The molecular structure of the title compound with the atom numbering scheme. Displacement ellipsoids are drawn at the 30% probability level. H atoms are presented as a small spheres of arbitrary radius.

**Fig. 2.**
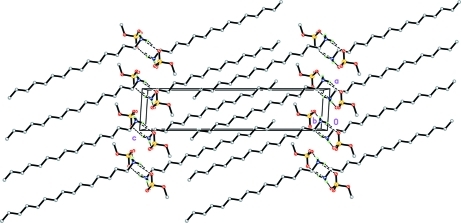
N—H···O interactions and intramolecular (dotted lines) in the crystal structure of the title compound. [Symmetry codes: (i) -x + 1, -y + 1, -z; (ii) x + 1, y, z]

## supplementary crystallographic information

### Comment

Long-chain *n*–alkylammonium halides are widely used as surfactants (Aratono *et al.*, 1998; Tornblom *et al.*, 2000) and as models for biological membranes (Ringsdorf *et al.*, 1988). They exhibit polymorphism at room temperature; solid-solid phase transitions occurred in *n*–alkylammonium chlorides (Terreros *et al.*, 2000). As a part of the studies on novel potential phase transition materials with the thermochemical properties such as *n*–alkylammonium chlorides, we report the crystal structure of the title compound (Fig. 1).

Atoms N1–C15 are coplanar in the title compound. The Space group of the title compound is P2(1)/m, however, the space group is P2(1)/c (Melanie Rademeyer,2009). The title compound has a symmetry plane, similarly, the n-pentadecylammonium bromide monohydrates has a symmetry axis. Furthermore, the S, O1, O2 and C16 are coplanar in the title compound.

The crystal packing (Fig. 2) is stabilized by one intermolecular N—H···O hydrogen bonds forming ionic pairs, and two other intramolecular N—H···O hydrogen bonds (Table 1).

### Experimental

*n*–Pentadecylammonium methyl sulfate was prepared by the addition of sulfuric acid to an methanol solution of *n*–pentadecylamine. The mixture was heated and stirred under reflux for 6 h. Single crystals suitable for *X*–ray diffraction were prepared by evaporation of the resulting solutionat room temperature. Analysis, calculated for C_16_H_37_NSO_4_ (Mr =339.53): C 56.60, H 10.98, N 4.13%; found: C 56.59, H 10.99, N 4.12%.

### Refinement

All the H atoms were placed in geometrically idealized positions and constrained to ride on their parent atoms, with methylene C—H distances of 0.97 Å, methyl C—H distances of 0.96 Å, N—H 0.89 Å and refined as riding on their parent atoms. The*U*_iso_(H) values were set at 1.2*U*_eq_ for the methylene H atoms and at 1.5*U*_eq_ for other H atoms.

### Crystal data

**Table:**

C_15_H_34_N^+^·CH_3_O_4_S^−^	*F*(000) = 376
*M**_r_* = 339.53	*D*_x_ = 1.139 Mg m^−^^3^
Monoclinic, *P*2_1_/*m*	Mo *K*α radiation, λ = 0.71073 Å
Hall symbol: -P 2yb	Cell parameters from 823 reflections
*a* = 5.4260 (5) Å	θ = 2.5–26.3°
*b* = 7.4981 (6) Å	µ = 0.18 mm^−^^1^
*c* = 24.376 (2) Å	*T* = 298 K
β = 93.557 (1)°	Acicular, colourless
*V* = 989.83 (15) Å^3^	0.31 × 0.30 × 0.28 mm
*Z* = 2	

### Data collection

**Table:**

Siemens SMART CCD area-detector diffractometer	1880 independent reflections
Radiation source: fine-focus sealed tube	1025 reflections with *I* > 2σ(*I*)
graphite	*R*_int_ = 0.062
Detector resolution: 10 pixels mm^-1^	θ_max_ = 25.0°, θ_min_ = 2.5°
phi and ω scans	*h* = −6→6
Absorption correction: multi-scan (*SADABS*; Sheldrick, 1996)	*k* = −8→8
*T*_min_ = 0.946, *T*_max_ = 0.951	*l* = −26→28
5243 measured reflections	

### Refinement

**Table:**

Refinement on *F*^2^	Primary atom site location: structure-invariant direct methods
Least-squares matrix: full	Secondary atom site location: difference Fourier map
*R*[*F*^2^ > 2σ(*F*^2^)] = 0.074	Hydrogen site location: inferred from neighbouring sites
*wR*(*F*^2^) = 0.228	H-atom parameters constrained
*S* = 1.06	*w* = 1/[σ^2^(*F*_o_^2^) + (0.1131*P*)^2^] where *P* = (*F*_o_^2^ + 2*F*_c_^2^)/3
1880 reflections	(Δ/σ)_max_ = 0.001
130 parameters	Δρ_max_ = 0.43 e Å^−^^3^
0 restraints	Δρ_min_ = −0.24 e Å^−^^3^

### Special details

**Table:**

Geometry. All e.s.d.'s (except the e.s.d. in the dihedral angle between two l.s. planes) are estimated using the full covariance matrix. The cell e.s.d.'s are taken into account individually in the estimation of e.s.d.'s in distances, angles and torsion angles; correlations between e.s.d.'s in cell parameters are only used when they are defined by crystal symmetry. An approximate (isotropic) treatment of cell e.s.d.'s is used for estimating e.s.d.'s involving l.s. planes.
Refinement. Refinement of *F*^2^ against ALL reflections. The weighted *R*-factor *wR* and goodness of fit *S* are based on *F*^2^, conventional *R*-factors *R* are based on *F*, with *F* set to zero for negative *F*^2^. The threshold expression of *F*^2^ > σ(*F*^2^) is used only for calculating *R*-factors(gt) *etc*. and is not relevant to the choice of reflections for refinement. *R*-factors based on *F*^2^ are statistically about twice as large as those based on *F*, and *R*- factors based on ALL data will be even larger.

### Fractional atomic coordinates and isotropic or equivalent isotropic displacement parameters (Å^2^)

**Table:**

	*x*	*y*	*z*	*U*_iso_*/*U*_eq_	
S1	0.3365 (3)	0.7500	0.07723 (6)	0.0623 (6)	
O1	0.3834 (8)	0.7500	0.14136 (16)	0.0806 (13)	
O2	0.0747 (8)	0.7500	0.07105 (18)	0.1042 (16)	
O3	0.4507 (5)	0.5923 (3)	0.05732 (12)	0.0784 (10)	
N1	1.2215 (8)	0.2500	0.03004 (18)	0.0562 (12)	
H1A	1.1749	0.2500	−0.0056	0.084*	
H1B	1.3113	0.3469	0.0381	0.084*	
C1	0.9998 (10)	0.2500	0.0626 (2)	0.0559 (14)	
H1	0.9012	0.3544	0.0527	0.067*	
C2	1.0542 (10)	0.2500	0.1226 (2)	0.0582 (14)	
H2	1.1521	0.3545	0.1327	0.070*	
C3	0.8258 (10)	0.2500	0.1545 (2)	0.0601 (14)	
H3	0.7285	0.3543	0.1437	0.072*	
C4	0.8663 (11)	0.2500	0.2159 (2)	0.0702 (16)	
H4	0.9641	0.3542	0.2265	0.084*	
C5	0.6433 (11)	0.2500	0.2481 (2)	0.0720 (17)	
H5	0.5458	0.3541	0.2374	0.086*	
C6	0.6793 (13)	0.2500	0.3087 (3)	0.087 (2)	
H6	0.7782	0.3539	0.3190	0.104*	
C7	0.4670 (12)	0.2500	0.3429 (2)	0.0811 (19)	
H7	0.3682	0.3538	0.3325	0.097*	
C8	0.5000 (14)	0.2500	0.4025 (3)	0.098 (2)	
H8	0.5995	0.3537	0.4127	0.117*	
C9	0.2936 (12)	0.2500	0.4378 (2)	0.085 (2)	
H9	0.1941	0.3538	0.4277	0.102*	
C10	0.3271 (14)	0.2500	0.4972 (3)	0.101 (2)	
H10	0.4269	0.3537	0.5072	0.122*	
C11	0.1238 (13)	0.2500	0.5329 (3)	0.089 (2)	
H11	0.0241	0.3537	0.5228	0.106*	
C12	0.1563 (14)	0.2500	0.5919 (3)	0.106 (2)	
H12	0.2565	0.3536	0.6018	0.127*	
C13	−0.0447 (14)	0.2500	0.6282 (3)	0.092 (2)	
H13	−0.1449	0.3536	0.6184	0.111*	
C14	−0.0117 (16)	0.2500	0.6873 (3)	0.116 (3)	
H14	0.0882	0.3537	0.6973	0.139*	
C15	−0.2143 (16)	0.2500	0.7228 (3)	0.113 (3)	
H15C	−0.1514	0.2500	0.7605	0.169*	
H15D	−0.3134	0.3545	0.7157	0.169*	
C16	0.6344 (14)	0.7500	0.1633 (3)	0.096 (2)	
H16A	0.6379	0.7500	0.2028	0.144*	
H16B	0.7168	0.8545	0.1510	0.144*	

### Atomic displacement parameters (Å^2^)

**Table:**

	*U*^11^	*U*^22^	*U*^33^	*U*^12^	*U*^13^	*U*^23^
S1	0.0630 (10)	0.0772 (11)	0.0489 (9)	0.000	0.0208 (7)	0.000
O1	0.077 (3)	0.116 (3)	0.050 (3)	0.000	0.015 (2)	0.000
O2	0.066 (3)	0.177 (5)	0.071 (3)	0.000	0.021 (2)	0.000
O3	0.096 (2)	0.0590 (16)	0.083 (2)	−0.0101 (15)	0.0296 (18)	−0.0184 (14)
N1	0.054 (3)	0.056 (3)	0.060 (3)	0.000	0.017 (2)	0.000
C1	0.052 (3)	0.063 (3)	0.054 (4)	0.000	0.017 (3)	0.000
C2	0.057 (3)	0.065 (3)	0.054 (4)	0.000	0.012 (3)	0.000
C3	0.060 (3)	0.065 (3)	0.057 (4)	0.000	0.018 (3)	0.000
C4	0.072 (4)	0.081 (4)	0.060 (4)	0.000	0.020 (3)	0.000
C5	0.074 (4)	0.087 (4)	0.056 (4)	0.000	0.023 (3)	0.000
C6	0.083 (5)	0.112 (5)	0.067 (4)	0.000	0.026 (3)	0.000
C7	0.080 (5)	0.104 (5)	0.063 (4)	0.000	0.028 (3)	0.000
C8	0.089 (5)	0.136 (6)	0.071 (5)	0.000	0.027 (4)	0.000
C9	0.082 (5)	0.111 (5)	0.064 (5)	0.000	0.027 (4)	0.000
C10	0.095 (6)	0.139 (6)	0.073 (5)	0.000	0.030 (4)	0.000
C11	0.090 (5)	0.115 (5)	0.064 (5)	0.000	0.026 (4)	0.000
C12	0.104 (6)	0.143 (7)	0.074 (5)	0.000	0.034 (4)	0.000
C13	0.100 (5)	0.116 (6)	0.064 (5)	0.000	0.029 (4)	0.000
C14	0.121 (7)	0.152 (7)	0.077 (6)	0.000	0.041 (5)	0.000
C15	0.128 (7)	0.135 (7)	0.081 (6)	0.000	0.043 (5)	0.000
C16	0.110 (6)	0.106 (5)	0.072 (5)	0.000	0.013 (4)	0.000

### Geometric parameters (Å, °)

**Table:**

S1—O2	1.419 (5)	C7—C8	1.453 (8)
S1—O3^i^	1.434 (3)	C7—H7	0.9700
S1—O3	1.434 (3)	C8—C9	1.454 (8)
S1—O1	1.568 (4)	C8—H8	0.9700
O1—C16	1.432 (8)	C9—C10	1.450 (8)
N1—C1	1.481 (6)	C9—H9	0.9700
N1—H1A	0.8900	C10—C11	1.445 (8)
N1—H1B	0.8900	C10—H10	0.9700
C1—C2	1.474 (7)	C11—C12	1.439 (9)
C1—H1	0.9700	C11—H11	0.9700
C2—C3	1.503 (7)	C12—C13	1.447 (9)
C2—H2	0.9700	C12—H12	0.9700
C3—C4	1.500 (7)	C13—C14	1.440 (8)
C3—H3	0.9700	C13—H13	0.9700
C4—C5	1.483 (7)	C14—C15	1.441 (9)
C4—H4	0.9700	C14—H14	0.9700
C5—C6	1.478 (8)	C15—H15C	0.9600
C5—H5	0.9700	C15—H15D	0.9600
C6—C7	1.462 (8)	C16—H16A	0.9600
C6—H6	0.9700	C16—H16B	0.9600
			
O2—S1—O3^i^	114.50 (15)	C8—C7—H7	107.0
O2—S1—O3	114.50 (15)	C6—C7—H7	107.1
O3^i^—S1—O3	111.2 (2)	C7—C8—C9	122.7 (7)
O2—S1—O1	101.8 (3)	C7—C8—H8	106.7
O3^i^—S1—O1	106.91 (15)	C9—C8—H8	106.6
O3—S1—O1	106.91 (15)	C10—C9—C8	122.6 (6)
C16—O1—S1	117.7 (4)	C10—C9—H9	106.7
C1—N1—H1A	109.4	C8—C9—H9	106.7
C1—N1—H1B	109.5	C11—C10—C9	123.2 (7)
H1A—N1—H1B	109.5	C11—C10—H10	106.5
C2—C1—N1	114.3 (4)	C9—C10—H10	106.5
C2—C1—H1	108.6	C12—C11—C10	123.4 (7)
N1—C1—H1	108.7	C12—C11—H11	106.5
C1—C2—C3	113.1 (4)	C10—C11—H11	106.4
C1—C2—H2	109.0	C11—C12—C13	124.2 (7)
C3—C2—H2	108.9	C11—C12—H12	106.3
C4—C3—C2	116.2 (5)	C13—C12—H12	106.3
C4—C3—H3	108.3	C14—C13—C12	124.1 (7)
C2—C3—H3	108.2	C14—C13—H13	106.3
C5—C4—C3	117.0 (5)	C12—C13—H13	106.3
C5—C4—H4	108.0	C13—C14—C15	123.2 (8)
C3—C4—H4	108.0	C13—C14—H14	106.5
C6—C5—C4	117.9 (5)	C15—C14—H14	106.5
C6—C5—H5	107.8	C14—C15—H15C	109.6
C4—C5—H5	107.8	C14—C15—H15D	109.4
C7—C6—C5	120.6 (6)	H15C—C15—H15D	109.5
C7—C6—H6	107.2	O1—C16—H16A	109.5
C5—C6—H6	107.2	O1—C16—H16B	109.5
C8—C7—C6	121.1 (6)	H16A—C16—H16B	109.5
			
O2—S1—O1—C16	180.000 (1)	C5—C6—C7—C8	180.000 (3)
O3^i^—S1—O1—C16	−59.57 (14)	C6—C7—C8—C9	180.000 (3)
O3—S1—O1—C16	59.57 (14)	C7—C8—C9—C10	180.000 (4)
N1—C1—C2—C3	180.0	C8—C9—C10—C11	180.000 (3)
C1—C2—C3—C4	180.000 (1)	C9—C10—C11—C12	180.000 (4)
C2—C3—C4—C5	180.000 (1)	C10—C11—C12—C13	180.000 (5)
C3—C4—C5—C6	180.000 (2)	C11—C12—C13—C14	180.000 (6)
C4—C5—C6—C7	180.000 (2)	C12—C13—C14—C15	180.000 (6)

Symmetry codes: (i) *x*, −*y*+3/2, *z*.

### Hydrogen-bond geometry (Å, °)

**Table:**

*D*—H···*A*	*D*—H	H···*A*	*D*···*A*	*D*—H···*A*
N1—H1A···O2^ii^	0.89	2.03	2.857 (6)	155.
N1—H1B···O3^iii^	0.89	2.03	2.911 (3)	169.

Symmetry codes: (ii) −*x*+1, −*y*+1, −*z*; (iii) *x*+1, *y*, *z*.
